# Investigating the impact of polymorphisms in the *ANKK1* and *DRD2* genes on oral health-related quality of life in male patients with temporomandibular disorders

**DOI:** 10.3389/froh.2025.1561781

**Published:** 2025-06-20

**Authors:** Samantha Schaffer Pugsley Baratto, Allan Abuabara, Débora Cristina Cardozo Bueno, Thalita de Paris Matos, Camila Paiva Perin, Gisele Maria Correr, César Penazzo Lepri, Christian Kirschneck, Flares Baratto-Filho, Erika Calvano Küchler

**Affiliations:** ^1^ School of Dentistry, University Center UniDomBosco, Curitiba, Brazil; ^2^ Department of Dentistry, University of the Joinville Region—Univille, Joinville, Brazil; ^3^ School of Dentistry, Tuiuti University of Paraná, Curitiba, Brazil; ^4^ School of Dentistry, Paraná Federal University, Curitiba, Brazil; ^5^ School of Dentistry, Uberaba University, Uberaba, Brazil; ^6^ Department of Orthodontics, University Hospital Bonn, Medical Faculty, Bonn, Germany

**Keywords:** quality of life, genes, pain, temporomandibular joint disorders, oral health

## Abstract

**Introduction:**

Previous studies have reported that genetic polymorphisms may impact the signs and symptoms of temporomandibular disorder (TMD). Therefore, this study aimed to investigate the association between polymorphisms in the *Dopamine Receptor D2* (*DRD2*) and *Ankyrin Repeat and Kinase Domain Containing 1* (*ANKK1*) genes and oral health-related quality of life of male patients with TMD.

**Methods:**

This cross-sectional study included construction workers with at least one sign or symptom of TMD. The reduced version of the Oral Health Impact Profile questionnaire (OHIP-14) was used to assess oral health-related quality of life. Genomic DNA was used to genotype genetic polymorphisms in the locus 11q22-q23, one in *ANKK1* (rs1800497) and two in *DRD2* (rs6275 and rs6276), using real-time polymerase chain reaction. The total OHIP-14 score and those for each domain were compared among the genotypes using the Kruskal–Wallis test and Dunn's test in the genotypic co-dominant model. The Mann–Whitney test was used in the recessive model (alpha = 0.05).

**Results:**

The sample included a total of 114 male patients. OHIP-14 total score ranged from 0 to 33. Chronic pain (87.7%), followed by disc displacement (38.2%), was the most common sign and symptom observed. All the genetic polymorphisms assessed were within the Hardy–Weinberg equilibrium. The “Handicap” domain (D6) was statistically associated with the genetic polymorphism rs1800497 in *ANKK1* (*p* = 0.008). The genetic polymorphism rs1800497 Taq1A in *DRD2/ANKK1* was associated with oral health-related quality of life, as measured by the handicap domain in OHIP-14, in male patients with TMD.

**Discussion:**

This study showed that genetic polymorphisms can negatively impact the oral health-related quality of life, as measured by the handicap domain of the OHIP-14. The physical and emotional condition of patients, together with biological pathways, should receive more attention in future studies, and personalized treatment plans should be created to improve patients' quality of life.

## 1 Introduction

Temporomandibular disorders (TMD) are defined as a set of diseases and disorders that are related to alterations in the structure, function, or physiology of the masticatory system and that may be associated with other systemic and comorbid medical conditions (1). Typical symptoms and signs of TMD include local pain, limitations of masticatory function, noises, and deviations of the mandible (2). The overall prevalence of TMD is approximately 31% for adults/elderly in the general population, depending on the characteristics of the studied group (3). The “Diagnostic Criteria for Research in Temporomandibular Disorders” (RDC/TMD) is a standardized tool for the assessment and classification of TMD. One important aspect of this instrument is that it is based on the biopsychosocial model of pain and evaluates factors related to a physical disorder in Axis I, while factors related to a psychosocial disorder are evaluated on Axis II (4).

In 2004, the National Institutes of Health (NIH) called for research proposals to conduct a prospective cohort study aimed at identifying the incidence of craniofacial pain and dysfunction, along with its associated risk factors (5). The NIH subsequently funded a project entitled Orofacial Pain: Prospective Evaluation and Risk Assessment (OPPERA). Thus, several studies have deepened the investigation into the etiology of TMD and aspects related to orofacial pain. These studies have shown that the origin of TMD extends far beyond local factors such as occlusal or masticatory dysfunction, third molar extraction, facial trauma, and degenerative arthritis. Growing evidence supports a multifactorial etiology, encompassing biopsychosocial and genetic factors, including age, sex, stress, depression, somatic symptoms, and psychological distress phenotypes (5–9).

Studies have been investigating quality of life in association with TMD (10, 11). According to the World Health Organization (WHO) (12), quality of life is defined as a person's perception of their position in life according to the value systems of the society in which they live (13). A previous systematic review showed that patients with TMD have worse quality of life than non-TMD patients (11). Oral health-related quality of life (OHRQoL) is a multidimensional construct that covers a subjective assessment of the oral health, functional wellbeing, emotional wellbeing, expectations and satisfaction of a patient (12).

Data from OPPERA indicated that genetic variations in candidate genes may contribute to the development of painful TMD (5, 7–9). Recent studies suggest that molecular factors influence the course of transcriptional activity in affected tissues, ultimately determining whether pain resolves or develops into a chronic condition (7). In addition, insights into genetic predisposition in quality of life have been gained in recent years, including the creation of the Consortium for Genetics and Quality of Life Research (GeneQoL) (14). Two candidate genes for quality of life research are *Dopamine Receptor D2* (*DRD2*) and *Ankyrin Repeat and Kinase Domain Containing 1* (*ANKK1*). Both genes are located adjacent to each other on chromosome 11 (11q23.1) (15). They are candidate genes as dopamine is a well-known important endogenous catecholamine that plays a key role in controlling emotion.

The genetic polymorphism rs1800497 in *DRD2/ANKK1* can reduce the expression of the dopamine receptor (DRD2), affecting the dopaminergic pathway (16, 17). *DRD2* was targeted as a candidate gene due to the evidence of its association with an individual’s pain perception (18). Our previous study reported that genetic polymorphisms within *DRD2* and *ANKK1* may have impacted TMD signs and symptoms among a group of male construction workers (10). Our hypothesis is that these two genes have a pleiotropic effect, i.e., when a single gene has a genetic effect on several phenotypic traits. Thus, in this study, we evaluated the role of three well-known genetic polymorphisms on the locus 11q22-q23 (*DRD2/ANKK1*) in the OHRQoL of these male patients with signs and symptoms of TMD.

This study raises the hypothesis that genetic polymorphisms in the *DRD2*/*ANKK1* gene cluster may contribute to the signs and symptoms of TMD. In contrast to previous research, our study investigates this relationship within a specific occupational group, male construction workers, who are particularly susceptible to musculoskeletal disorders. In addition, TMD-related pain in men remains a largely underexplored area. Therefore, the aim of this study was to examine, for the first time, the association between genetic polymorphisms in the *ANKK1* and *DRD2* genes and oral health-related quality of life in male patients with TMD.

## 2 Methods

### 2.1 Ethical aspects, sample description, and study design

This cross-sectional study was performed in the dental clinic of the Social Service of the Civil Construction Industry, a non-profit organization connected to the employer's union of the civil construction industry. The organization aims to promote health and safety in the workplace environment for construction workers. This sample was previously described in Baratto et al. (10). Only patients with good oral health were included.

Only patients with no major oral health conditions and at least one sign or symptom of TMD were included. All the participants agreed to participate and signed the free and informed consent form, which had previously been explained orally. Their personal data were recorded by interviewers only after their approval. The study protocol was accepted by the local human research ethics committee (number 2.802.708) that approved the study.

Construction workers from different trades were consecutively screened and included in the study from 2018 to 2019. Moreover, the Strengthening the Reporting of Genetic Association Studies (STREGA) checklist was followed when designing this study and reporting the results (19).

Illiterate workers and functional illiterates, i.e., individuals who were not able to understand and express themselves in the written form, were also not included in the screening process. Women and individuals who did not have any TMD signs or symptoms were excluded. All the construction laborers were men aged 18 years or older who had not reported the use of analgesic medications or antibiotics within the preceding 6 months and who were in good physical health.

### 2.2 Screening of patients and TMD examination

During the dental treatment appointment, the patients were recruited. The patients were invited to participate in the study and answered the questionnaire. The clinical (phenotypic) examination was made by a senior dentist who had experience in diagnosing TMD. The examiner was also previously trained and calibrated according to Axis I of the RDC/TMD. Axis I provides diagnostic criteria for three groups of disorders:

• myofascial pain (with or without mouth opening limitation),
• disc displacements and inflammatory conditions by side (with or without reduction),
• inflammatory conditions by side (arthralgia, osteoarthritis, and osteoarthrosis).

Axis II of the RDC/TMD was filled out by the participants and measures both pain levels and depressive symptoms. Chronic pain was graded, ranging from 0 to IV (0, low incapacity; I, low intensity; II, high intensity; III, moderate limitation; and IV, severe limitation). For depression, non-specific physical symptoms including pain and non-specific physical symptoms excluding pain were classified as low, moderate, or severe.

### 2.3 Oral health-related quality of life evaluation

The reduced version of the Oral Health Impact Profile questionnaire (OHIP-14), validated for the Portuguese language, was used in the present study. It consists of 14 questions, two from each of the seven domains of the instrument, which are as follows: domain 1, functional limitation; domain 2, physical pain; domain 3, psychological discomfort; domain 4, physical disability; domain 5, psychological disability; domain 6, handicap; and domain 7, social disability.

In this questionnaire, for each question, five answers are possible: never, seldom, sometimes, recurrently, or always. These answers are scored as 0, 1, 2, 3, and 4 points, respectively. The combined answers give a total score (OHIP-total) that can range from 0 to 56. A higher total score shows a larger negative impact on the oral health-related quality of life of the patient. The score in each domain varies from 0 to 8, with a higher score showing greater impairment in that domain.

### 2.4 Laboratory analysis

Deoxyribonucleic acid (DNA) isolated from oral cells was also collected during the dental examination (19). These stored DNA samples were used for the genotyping analysis.

The selection of the genetic polymorphisms was based on their previous association with pain traits, bruxism, and depression. The genetic polymorphisms rs1800497, rs6275, and rs6276 within *DRD1/ANKK1* (11q23.2) were screened and selected for investigation. Their characteristics are shown in Table 1. The genotyping was performed blinded using real-time polymerase chain reaction (StepOnePlus™ Real-time PCR System) using the TaqMan™ assay (Applied Biosystems, Foster City, CA, USA). There was a total volume of 3 μL per reaction (4 ng DNA/reaction, 1.5 μL Taqman PCR master mix, 0.125 SNP assay; Applied Biosystems, Foster City, CA). Each thermal cycle was set as follows: holding cycle of 95°C (10 min), and 40 amplification cycles of 92°C (15 s) and 60°C (1 min). Each 96-well plate had two negative controls. An internal consistency test was also conducted by randomly rerunning 10% of all the samples to reduce potential bias and this resulted in 100% agreement.

**Table 1:** The studied genes and genetic polymorphisms.

Genetic variant and base change	Function	Previously published phenotype-genotype associations
rs1800497 (C/T)^a^	Coding region-missense	Associated with migraine susceptibility (20) and bruxism (21), moderates the effect of stressful life events on depressive symptoms (22), and increases the risk of a suicide attempt (23)
rs6275 (A/G)	Coding region-synonymous variant	Associated with acute pain severity after a motor vehicle collision (24), and with tooth grinding (21), TMD in teenagers (25), and migraine susceptibility (20)
rs6276 (C/T)	UTR variant	Associated with acute pain severity after a motor vehicle collision (24), and with tooth grinding (21), TMD (10), and insight problem solving (26)

C/T, heterozygous, with one copy of the cytosine base and one copy of the thymine base; A/G, heterozygous, with one copy of the adenine base and one copy of the guanine base; TMD, temporomandibular disorder; UTR, untranslated region.

^a^ Also known as DRD2/ANKK1 Taq1A.

### 2.5 Statistical analysis

The sample size calculation for this preliminary study was performed assuming a mean difference of 1.0 among genotypes using an alpha of 5% and a power of 80%. Thus, a minimum sample size of 110 was required.

The Shapiro–Wilk test was used to test the normality of the data. The OHIP-14 data were presented as a median and range with the minimum and maximum values. The total OHIP-14 score and that of each domain were compared among the genotypes using the Kruskal–Wallis test and Dunn's test in the genotypic co-dominant model. The Mann–Whitney test was used in the recessive model. Spearman's correlation coefficient was used to evaluate the correlation between age in years and OHIP-14 total and domain scores. Values with a probability over 95% (alpha = 0.05) were deemed to be statistically significant. For each variant, samples that did not result in successful amplification were not included in the corresponding statistical analysis to avoid bias from incomplete genotyping data. The standard chi-square test was used to test for deviation from the Hardy–Weinberg equilibrium. A population is considered to be in Hardy–Weinberg equilibrium for a specific gene when five conditions are met: random mating, no mutations, no gene flow, no natural selection, and a sufficiently large population size. When these criteria are fulfilled, allele frequencies are expected to remain stable over time (27). All analyses were performed using Prism 8 software (GraphPad Software Inc., San Diego, CA, USA).

## 3 Results

**Figure 1:**
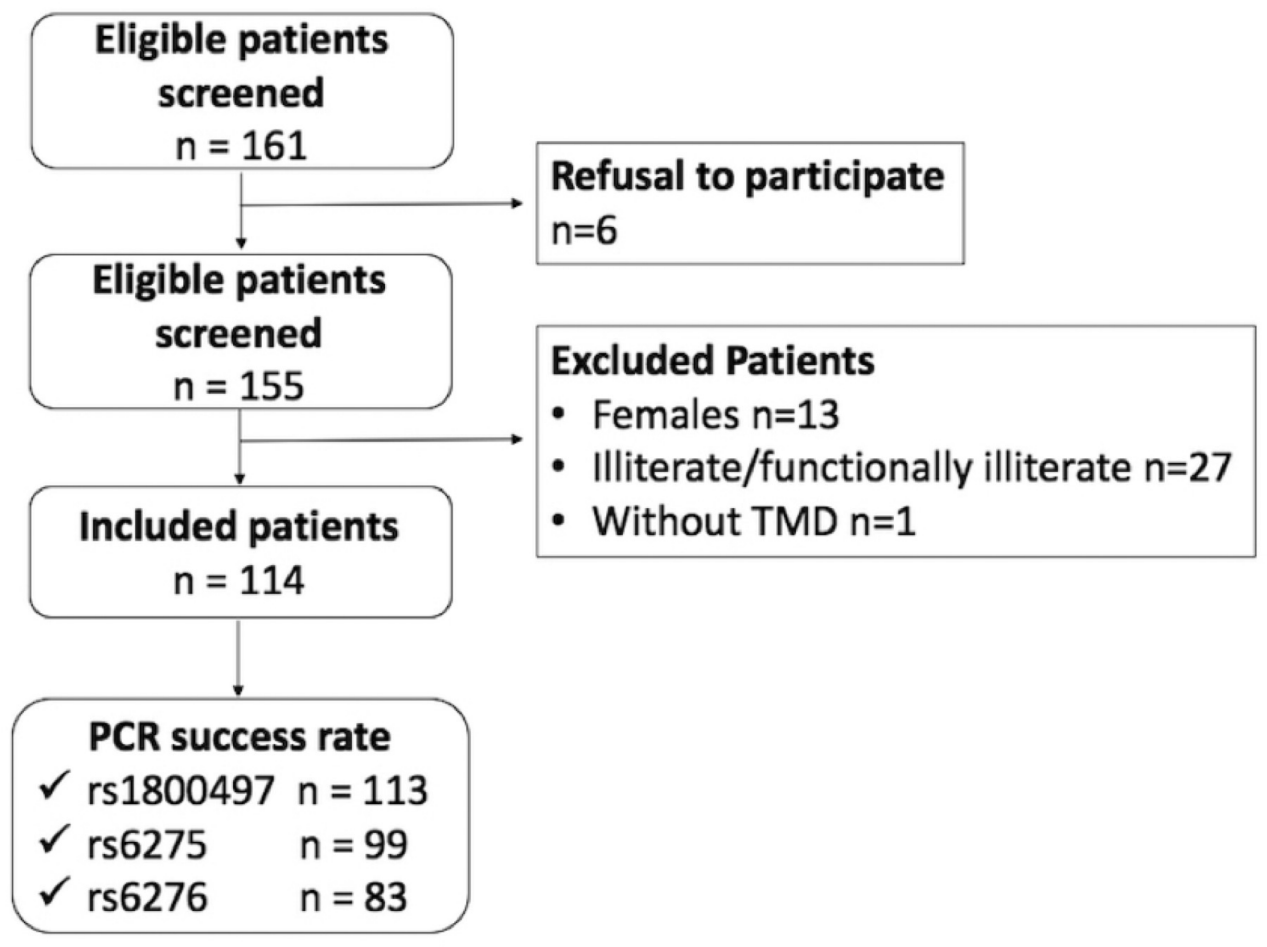
Flowchart of the participant recruitment process.

We included a total of 114 adult male patients with TMD. A flowchart illustrating the participant recruitment process is presented in Figure 1. Their age ranged from 19 to 77 years old. The mean age in years was 38.2 (standard deviation = 11.7 years). The distribution of TMD characteristics in the study sample is shown in Table 2. Age was not correlated (Spearman's correlation test) with OHIP-14 domain scores (OHIP-14 total *r*^2^ = 0.235; functional limitation *r*^2^ = 0.167; physical pain *r*^2^ = 0.239; physiological discomfort *r*^2^ = 0.093; physical disability *r*^2^ = 0.176; physiological disability *r*^2^ = 0.171; handicap *r*^2^ = 0.096; and social disability *r*^2^ = 0.169).

**Table 2:** Demographic and clinical characteristics of the participants according to TMD signs and symptoms (RDC/TMD Axes I and II).

Demographics of patient participants	
Male (*n*) (%)	114 (100%)
Age (range, mean, and SD)	19–77 (38.2 ± 11.7)
Non-smokers	114 (100%)
TMD signs and symptoms	Frequency (*n*) (%)
Myofascial pain	7 (6%)
Disc displacement	44 (39%)
Joint inflammation	31 (27%)
Chronic pain	100 (88%)
Depression	15 (13%)
Non-specific physical symptoms including pain^a^	
Normal	103 (90%)
Mild	10 (9%)
Non-specific physical symptoms excluding pain^a^	
Normal	103 (90%)
Mild	10 (9%)

TMD, temporomandibular disorder; RDC/TMD, Diagnostic Criteria for Research in Temporomandibular Disorders; SD, standard deviation.

^a^ No severe cases were found. One patient did not answer.

Table 3 presents the genotype frequencies for rs6276, rs6275, and rs1800497 in the studied population, indicating that all genetic polymorphisms are in Hardy–Weinberg equilibrium. A chi-square value greater than 3.841 (*α* = 0.05, degrees of freedom = 1) would indicate a deviation from equilibrium, whereas lower values suggest a good fit.

**Table 3:** Genotype frequency distribution.

Variant	Genotype frequency (*n*)			HWE^chi-square^
rs1800497	TT = 6	CT = 47	CC = 60	0.688
rs6275	AA = 13	AG = 52	GG = 34	0.99
rs6276	AA = 17	AG = 40	GG = 26	0.050

HWE, Hardy–Weinberg equilibrium; TT, homozygous for the thymine base; AA, homozygous for the adenine base; CC, homozygous for the cytosine base; GG, homozygous for the guanine base; CT, heterozygous, carrying one copy of the cytosine base and one copy of the thymine base; AG, heterozygous, carrying one copy of the adenine base and one copy of the guanine base.

The total OHIP-14 scores ranged from 0 to 33. The functional limitation domain scores ranged from 0 to 6, with the physical pain, physiological discomfort, and physical disability domain scores ranging from 0 to 5, 0 to 8, and 0 to 8, respectively. Furthermore, the handicap and social disability domain scores ranged from 0 to 4 and 0 to 6, respectively. The associations between the genotypes in the studied genetic polymorphisms and OHRQoL (total OHIP-14 and OHIP-14 domain scores) are presented in Table 4. The handicap domain score was statistically associated with rs1800497, with individuals carrying the CC genotype having a better OHRQoL than individuals carrying CT (*p* = 0.008). In the recessive analysis (CC vs. CT + TT), individuals carrying CC also had a better score in the handicap domain than individuals carrying at least one T allele (CT + TT) (*p* = 0.016). Thus, individuals with the CC genotype had a more positive perception of the handicap domain.

**Table 4:** Comparison of the OHIP-14 total score and the OHIP-14 domain scores (D) according to genotype.

Genotype	D1	*p*-value	D2	*p*-value	D3	*p*-Value	D4	*p*-value	D5	*p*-value	D6	*p*-value	D7	*p*-value	Total OHIP-14	*p*-value
rs1800497																
CC	0 (0–4)	0.654	1.5 (0–3)	0.179	2 (0–3)	0.921	0 (0–3)	0.721	0 (0–3)	0.130	0 (0–0)^a^	0.008*	0 (0–3)	0.692	5.5 (2–15)	0.703
CT	0 (0–6)		0 (0–5)		2 (0–8)		1 (0–8)		1 (0–6)		1 (0–4)^b^		0 (0–6)		6 (0–33)	
TT	0 (0–6)		0 (0–4)		2 (0–8)		2 (0–8)		1 (0–6)		0 (0–4)^a,b^		0 (0–3)		5 (0–28)	
rs6275																
CC	0 (0–6)	0.557	0 (0–4)	0.343	2 (0–8)	0.996	0 (0–6)	0.197	1 (0–4)	0.916	0 (0–2)	0.474	0 (0–2)	0.147	4 (0–28)	0.425
CT	0 (0–6)		0 (0–5)		2 (0–8)		0 (0–7)		1 (0–6)		1 (0–4)		0 (0–6)		4.5 (0–33)	
TT	0 (0–4)		1 (0–4)		2 (0–6)		0 (0–6)		1 (0–6)		0 (0–4)		0 (0–3)		6 (0–20)	
rs6276																
AA	0 (0–6)	0.726	0 (0–4)	0.124	2 (0–8)	0.906	0 (0–6)	0.884	1 (0–4)	0.412	0 (0–2)	0.617	0 (0–2)	0.551	4 (0–28)	0.742
AG	0 (0–4)		0 (0–4)		2 (0–8)		0 (0–7)		0.5 (0–6)		0 (0–4)		0 (0–6)		5 (0–33)	
GG	0 (0–2)		1 (0–4)		1.5 (0–6)		0 (0–6)		1.5 (0–6)		1 (0–4)		0 (0–3)		5.5 (0–20)	

D1, functional limitation; D2, physical pain; D3, physiological discomfort; D4, physical disability; D5, physiological disability; D6, handicap; and D7, social disability; OHIP, Oral Health Impact Profile questionnaire; CC, homozygous for the cytosine base; CT, heterozygous, carrying one copy of the cytosine base and one copy of the thymine base; TT, homozygous for the thymine base; AA, homozygous for the adenine base; AG, heterozygous, carrying one copy of the adenine base and one copy of the guanine base; GG, homozygous for the guanine base.

Different letters indicate significant differences among genotypes.

* Statistical significance difference.

## 4 Discussion

Although the role of an individual's genetic background on patient-reported quality of life has attracted increased attention in recent years, the association between genetic polymorphisms and OHRQoL is still poorly explored, especially in patients with TMD. Several lines of evidence suggest that genes involved in dopamine transmission could be potential candidates for patient-reported quality of life and TMD due to their previously associated phenotypes (4, 10, 20–26, 28). Therefore, in the present study, we investigated whether genetic polymorphisms in genes involved in dopamine transmission could be potential candidates for OHRQoL in male construction workers with TMD.

In our study, our sample was composed only of male patients. It is well-known and documented in the literature that TMD is a condition that presents with sex differences. A recent systematic review reported the importance of sex in the development of TMD, with women at two times greater risk compared to men (29). Associations between specific polymorphisms in estrogen receptor alpha and beta genes and the presence or severity of TMD, particularly among women, are reported (30). However, inconsistent findings indicate the need for larger prospective studies to confirm these associations and better understand their clinical impact (30). In contrast, construction workers work in demanding physical conditions and experience a high risk of injuries. Their workplace environment is connected to their health and their wellbeing (31). Construction sites are usually recognized as workplaces associated with a high risk of injuries and poor health (31). Construction workers have an even greater risk of painful musculoskeletal strains and injuries (31). The pain severity is aggravated by their severe working environment (32). Therefore, this specific sample was selected in our study and the findings may not be applicable to the entire population, as the analysis focused exclusively on a specific gender and occupation. However, studies searching for biomarkers that could help improve the screening of individuals at a higher risk of an impact on their quality of life will allow for better individualized assistance.

*DRD2* was found to be a candidate gene for patient-reported quality of life. DRD2 was proposed due to the evidence of its role on pain perception (14). Therefore, in the present study, we explored, for the first time, the association between *DRD2/ANKK1* and OHRQoL. In our study, *DRD2/ANKK1* Taq1A (rs1800497) was statistically associated with OHRQoL. This genetic polymorphism is located in the gene that codes for *ANKK1* within a protein-coding region, leading to a Glu713-to-Lys (also known as E713K) substitution in the putative *ANKK1* protein. This polymorphism is near the termination codon of the *DRD2* gene, located on chromosome 11q22-q23. This is the most studied genetic polymorphism in a broad range of psychiatric traits and pain conditions (23, 33, 34). Jiang et al. found a remarkable relationship between the *DRD2* genotype, job stress, and sleep dysfunction among the Chinese Han population (35). Furthermore, the authors found that individuals with both the A1 allele of the *DRD2* gene and job stress were more likely to have sleep dysfunction (35). Carrying the T allele was associated with inferior OHRQoL, and similar results were observed, showing this polymorphism increases the risk of sleep dysfunction, among other conditions (35).

OHRQoL is important for dental clinical practice and dental research. OHRQoL questionnaires are useful instruments to predict psychological issues and estimate the impact of oral health on a patient’s quality of life in general (36). One of these questionnaires, the OHIP-14, is a short-form questionnaire that investigates the impact of oral health on daily activities, measuring the perception of the influence of oral health on the social sphere and the patient’s overall quality of life (36). OHIP-14 is intended for use in both clinical and research settings. Thus, this questionnaire/instrument was selected in our study. We also used the RDC/TMD to examine TMD, which is an instrument widely used in TMD research. This tool standardizes the assessment and classification of patients and is based on the biopsychosocial model of pain (37). Physical, psychological, and social factors contribute to the overall symptoms of TMD, and assessments of the physical (Axis I) and psychosocial aspects (Axis II) are crucial when studying TMD (37).

Our study has some important limitations that should be highlighted here. Although patients with untreated caries and periodontal disease were included, the study did not control for other confounding factors, such as the number of missing teeth. It is possible that some of the included patients may have had other oral health problems. It is also known that OHRQoL differs significantly by sleep status, smoking status, and alcohol consumption history (38), which were not evaluated here. Therefore, it is possible that some other factors influenced our results, as the OHIP-14 results could reflect other oral health problems. Another important limitation of this study is that the analyzed population consisted exclusively of adult male construction workers, which restricts the generalizability of the findings to other groups, such as women, younger individuals, or people with different occupations. While this choice is justified by the high occupational exposure to musculoskeletal pain risk factors in this group, it limits the sample's diversity and may reduce the broader understanding of genetic factors associated with TMD. Additionally, the absence of a control group hinders result comparisons and weakens the strength of the conclusions.

Previously, health and quality of life were directly associated with the medical model. More recently, the socio-environmental model guides many health strategies and investments. There is emerging evidence of a genetic basis for patient-reported quality of life. This study demonstrated that genetic polymorphisms may play a role in the negative impact of TMD on the handicap domain of OHRQoL. The handicap domain reflects the broader social and psychological disadvantages caused by oral health problems, highlighting how these conditions may interfere with an individual's overall wellbeing and societal participation. These findings suggest that genetic factors, alongside physical symptoms and emotional wellbeing, are important components influencing the overall burden of TMD. Such a comprehensive approach not only enhances our understanding of the underlying mechanisms but also informs more individualized and effective treatment strategies aimed at improving patients' quality of life. In the future, insight into the genetic background of patient-reported quality of life outcomes will allow us to explore new pathways for improving patients’ dental care and general health and allow us to identify patients who are susceptible to a poor quality of life. We will also be able to better prepare preventive strategies and specific treatment protocols according to the profile of the patient.

## 5 Conclusion

In conclusion, based on our study design and limitations, the results observed here suggest that the genetic polymorphism Taq1A (rs1800497) in *DRD2/ANKK1* is associated with oral health-related quality of life, as measured by the handicap domain in the OHIP-14, in male patients with TMD. However, future studies are necessary to investigate this topic.

## Data Availability

The original contributions presented in the study are publicly available. This data can be found here: 10.6084/m9.figshare.29294144.
